# Hydrogenation of pyrolysis gasoline by novel Ni-doped MOF derived catalysts from ZIF-8 and ZIF-67

**DOI:** 10.1038/s41598-022-24071-2

**Published:** 2022-11-12

**Authors:** Alireza Baghban, Hossein Ezedin Nejadian, Sajjad Habibzadeh, Farzin Zokaee Ashtiani

**Affiliations:** 1 Chemical Engineering Department, Amirkabir University of Technology (Tehran Polytechnic), Mahshahr Campus, Mahshahr, Iran; 2 Surface Reaction and Advanced Energy Materials Laboratory, Chemical Engineering Department, Amirkabir University of Technology (Tehran Polytechnic), Tehran, Iran; 3 Chemical Engineering Department, Amirkabir University of Technology, Tehran, Iran

**Keywords:** Chemistry, Energy science and technology, Engineering, Materials science

## Abstract

Pyrolysis gasoline is the valuable byproduct of the thermal breakdown of heavier oil fractions in an olefin unit with high aromatic content. To separate such aromatic components, firstly, this product should be hydrogenated. In this contribution, new nanostructure catalysts derived from the zeolitic metal–organic framework, namely ZIF-8 and ZIF-67, were used to investigate their hydrogenation capability. Owing to its great hydrogenation capability of Nickle, the structures of the ZIF-8 and ZIF-67 were improved by Nickle through in situ synthesis. Moreover, to enhance the pore size of catalysts and their electronic properties, the synthesized catalysts were pyrolyzed under nitrogen media at 450 °C, and five catalysts, namely Co/NC, ZnCo/NC, ZnNi/NC, CoNi/NC, and ZnCoNi/NC were created. Results indicated that the CoNi/NC showed a superior hydrogenation performance (69.5% conversion of total olefins) to others. In addition, the synthesized catalysts without the carbonization process had no conversion in the hydrogenation process because there is no active site in these structures. The current synthesized catalysts can compete with the costly Pt or Pd-based hydrogenation catalysts due to their high surface area and great electronic properties.

## Introduction

There are several uses for pyrolysis gasoline (PyGas), a valuable byproduct of the thermal breakdown of heavier oil fractions in an olefin unit. It contains aromatic hydrocarbons such as BTX, the derivatives and unsaturated components including mono- and di-olefins. In addition, PyGas because of a typically high-octane rating can be counted as a potential feedstock for aromatics production, and thereby gasoline blend stock^1–3^. However, it is indispensable to stabilize the unsaturated chemicals, which are gum agents, to sustain employing PyGas in the process. Namely, catalytic hydrogenation of PyGas has been the traditional way to stabilize such compounds^4,5^. It begins with hydrogenating di-olefins and alkenyl aromatics selectively at low temperatures without saturating with other unsaturated hydrocarbons; the resulting product may be utilized as a base for gasoline fuel blends. After that, the aromatics are entirely hydrogenated to remove any remaining sulfurs or olefins at high temperatures, which is the second step of the process^3,6^.

PyGas hydrogenation has recently attracted much interest because of the inexpensive cost, minimal toxicity, and strong gum resistance of supported nickel catalysts^7–9^. However, because of the massive Ni aggregates, Ni monometallic catalysts often have been suffering a poor catalytic efficiency. The PyGas hydrogenation process uses a variety of Ni-based bimetallic catalysts, including NiPt^10^, NiZn^11^, NiMo^12^, and NiCo^12^. It is demonstrated that bimetallic catalysts may outperform monometallic catalysts in different industrial processes^13–15^. When compared to Ni or Ru monometallic catalysts, NiRu bimetallic catalysts were shown to have synergistic effects in various catalytic processes^16–18^. These clusters or alloys are the results of the close contact between the bimetallic atoms.

In particular, the advancement of metal–organic frameworks (MOFs) hydrogen storage characteristics in recent years has made hydrogenation catalysis one of the most favorable uses of MOFs^19–23^. In the chemical industry, hydrogenation reactions are widely applied, and an effective hydrogenation catalyst is critical in these processes. Due to their distinct benefits, MOF materials may be used in a wide variety of hydrogenation processes. Furthermore, MOFs may be employed both as a support for highly scattered and critical hydrogen storage materials. As a result, MOFs in hydrogenation catalysis have an advantage over other catalysts. However, it is vital to mention that not all the MOF materials are suitable for catalytic hydrogenation. For the researchers to come up with plausible designs, they must take into account the unique properties of every MOF material and the different hydrogenation processes that occur.

Zeolitic imidazole frameworks (ZIFs) are one of the most stable MOFs in terms of thermal and chemical conditions and they typically consist of imidazolate-based ligands and a divalent metal (such as Zn^2+^ or Co^2+^)^24,25^. Many are microporous and, like zeolites, are used in applications that need just a narrow range of size and form. ZIFs and MOFs have recently been used to encapsulate catalytically active nanoparticles, resulting in catalysts for novel processes^26,27^.

It's clear that microporous ZIFs cannot perform as a suitable support for the hydrogenation of PyGas. Nevertheless, it has proven that the use of triethylamine in ZIF-8 and ZIF-67 synthesis can increase the production yield of these ZIFs up to 90% and give us a cost-effective synthesis also it can increase the mean pore diameter of the resulted ZIFs because the steric hindrance^28^. However, the mean pore diameter is still not favorable for catalytic applications. Also, the intrinsic metal species in ZIFs are not available as an active site in the pristine form because the Co^2+^ or the Zn^2+^ are fully bonded with imidazole by coordinative bonds. A carbonization step can increase the mean pore diameter even more beside the release of the intrinsic metal ions as metallic active sites during the pyrolysis step and turning 2-MIM into an N-doped carbon material which is promising for hydrogenation reaction because of its good electrical conductivity^29^. For example, Li et.al doped Ni in ZIF-67 (without using TEA) and after a pyrolysis step, they used it as an electrocatalyst for HER and named it CoNi/PC. The mean pore diameter of the resulted electrocatalyst was 3.95 nm and the electroconductivity of all samples increased after the carbonization step due to the formation of N-doped carbon support^30^.

In this study, new nanostructure catalysts derived from ZIF-8 and ZIF-67 were synthesized and used for the hydrogenation of PyGas. Moreover, Nickle was added to the ZIF structures during synthesis and improved their hydrogenation performance. To enhance the pore size distribution of the developed catalysts and also their electronic properties, the synthesized catalysts were pyrolyzed under nitrogen media at 450 °C and five catalysts namely Co/NC, CoZn/NC, NiZn/NC, NiCo/NC, and NiCoZn/NC were created and compared with the costly commercial Pd/Al_2_O_3_ catalyst in hydrogenation of olefins.

## Materials and methods

### Materials

Zn (NO_3_)_2_⋅6H_2_O (98%, Aldrich, Co (NO_3_)_2_⋅6H_2_O (98.0%, Aldrich) and Ni(NO_3_)_2_⋅6H_2_O (98%, Aldrich), 2-methylimidazole (2-MeIm, 98%, Aldrich), and triethylamine (TEA, 99.5%, Aldrich) were employed. On-site water deionization was provided by an in-house system.

### Synthesis of ZIF-67, ZIF-8, CoZn/ZIF, ZnNi/ZIF, CoNi/ZIF and ZnCoNi/ZIF

To synthesize ZIF-67, a metal: ligand: TEA molar ratio of 1:4:4 was used. 50 mL of DI water was used to dissolve 1.46 g of Co (NO_3_)_2_⋅6H_2_O. For the second solution, we used 1.64 g of 2-MeIm and 2.02 g of triethylamine in 50 ml of deionized water and stirred until the 2-MeIm and triethylamine were completely dissolved. The 2-MeIm/TEA solution was being swirled during the dropwise addition of the Co solution and it can be observed that the mixture quickly colored opaque purple. The suspension was then mixed for 12 h. Afterward, centrifugation was used to separate the synthesis mixture, and then the solid was re-dissolved in DI water after the supernatant was removed. This work was done three times and centrifugation time was 10 min for each stage. Finally, the ZIF suspension solid was recovered, dried in an oven at 100 °C for 12 h, and stored.

To synthesize ZIF-8, 1.49 g of Zn(NO_3_)_2_⋅6H_2_O was dissolved in 50 ml of DI water. TEA and 2-MeIm were added to 50 mL of DI water same as the ZIF-67 synthesis procedure and agitated until the solution was clear. Swirled 2-MeIm/TEA solution with Zn solution was stirred for 12 h after adding the Zn solution drop by drop to 2-MeIm/TEA. The liquid quickly becomes opaque white. The centrifuging and drying processes were done the same as ZIF-67 synthesis.

1.49 g Zn (NO_3_)_2_⋅6H_2_O with 1.46 g of Co (NO_3_)_2_⋅6H_2_O was each dissolved in 50 mL of DI water before proceeding with the experiment. A second solution was prepared by stirring 1.64 g of 2-MeIm (20 mmol) and 2.00 g of TEA (20 mmol) in 50 mL of DI water until they were dissolved. The 2-Me-Im/TEA solution was stirred when the Co/Zn solution was added drop by drop. The mixture instantly became opaque purple and continued to stir for 12 h. Centrifugation was used to separate the synthesis mixture, and then the solid was re-dissolved in DI water after the supernatant was removed. The centrifuging and drying processes were done the same as ZIF-67 synthesis.

In 50 mL of DI water, 1.49 g Zn (NO_3_)_2_⋅6H_2_O, and 1.45 g Ni (NO_3_)_2_⋅6H_2_O were dissolved. It was necessary to produce Ni/Zn solution by mixing Zn and Ni solutions. For the second solution, we used 1.64 g of 2-MeIm and 2 g of TEA in 50 ml of deionized water and stirred until the 2-MeIm and TEA were completely dissolved. After dropwise adding the Zn/Ni solution to the 2-MeIm/TEA solution and stirring it for 12 h, the liquid became opaque green. The centrifuging and drying processes were done the same as ZIF-67 synthesis.

Distilled water was used to dissolve both 1.46 g Co (NO_3_)_2_⋅6H_2_O and 1.45 g Ni (NO_3_)_2_⋅6H_2_O. Ni solution was added to the Co solution to produce Ni/Zn solution. For the second solution, we used 1.64 g of 2-MeIm and 2 g of TEA in 50 ml of deionized water and stirred until the 2-MeIm and TEA were completely dissolved. The 2-MeIm/TEA solution was swirled when the Co/Ni solution was added drop by drop, and the mixture quickly colored opaque purple. The prepared solution was stirred until 12 h and then the centrifuging and drying processes were done the same as ZIF-67 synthesis.

Separately, 50 mL of DI water was used to dissolve 1.49 g of Zn (NO_3_)_2_⋅6H_2_O, 1.46 g of Co(NO_3_)_2_⋅6H_2_O, and 1.45 g of Ni(NO_3_)_2_⋅6H_2_O. Afterward, we used 1.64 g of 2-MeIm and 2 g of TEA in 50 ml of deionized water and stirred until the 2-MeIm and TEA were completely dissolved. The 2-MeIm/TEA solution was swirled when the Zn/Co/Ni solution was added drop by drop, and the mixture quickly colored opaque purple. The prepared solution was stirred until 12 h and then the centrifuging and drying processes were done the same as ZIF-67 synthesis.

### Synthesizing ZIF-derived catalysts

**Figure 1:**
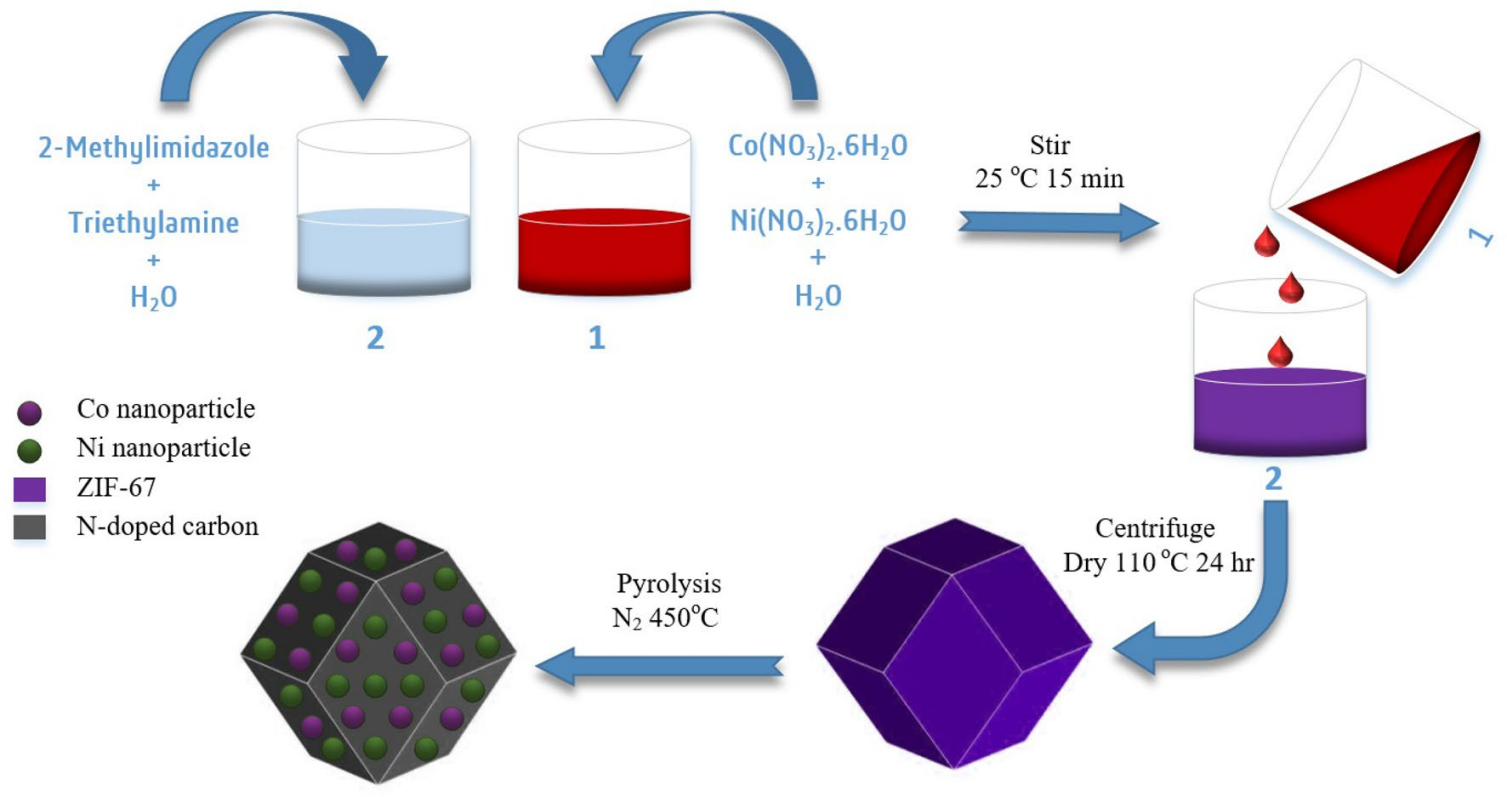
Schematic illustration of NiCo/NC catalyst synthesis.

Carbonization of the abovementioned ZIF catalysts was carried out to increase their chemical stabilities. ZIF-based catalysts were typically carbonized by first being put in a porcelain combustion boat and then transported to a tube furnace. We heated the samples with nitrogen at a flow rate of 5 °C/min, carbonized at 450 °C for 3 h. Figure 1 depicts the synthesis process of NiCo/NC.

### PyGas hydrogenation activity test

**Figure 2:**
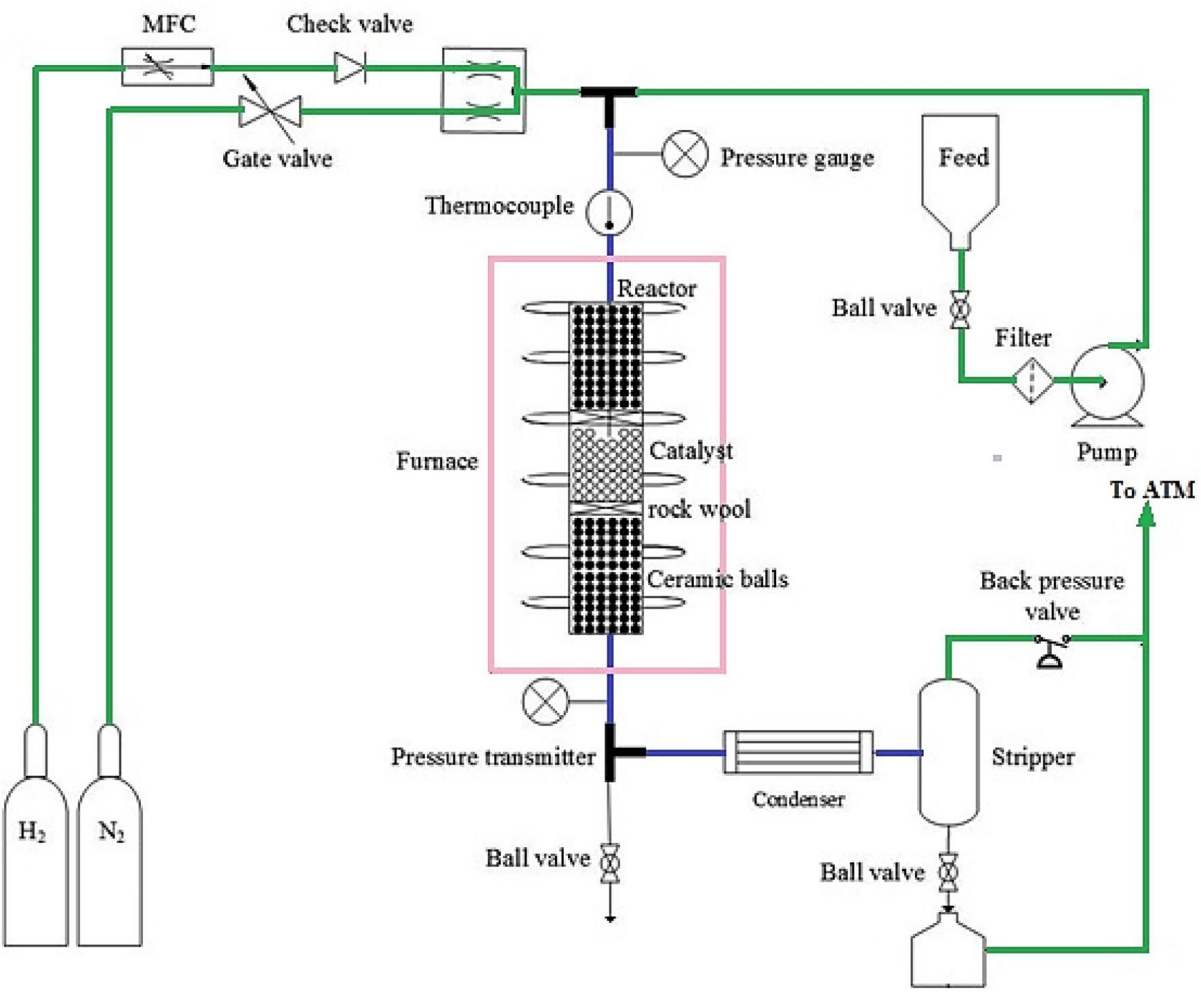
Schematic of the experimental set-up for hydrogenation of PyGas.

Under operating circumstances resembling the industrial hydrotreating of the PyGas unit, the catalytic activity test was performed in a fixed-bed reactor with an internal diameter of 20 mm and a height of 900 mm (see Fig. 2). Rock wool supported a bed containing 3 g of the catalyst in the reactor's constant temperature zone. The remaining areas of the reactor were filled with ceramic balls to avoid an improper distribution of temperature and mass transfer. An electric furnace with a cylindrical shape contained the reactor. The bed's temperature and pressure were measured using a thermocouple and a pressure transmitter. Additionally, the reactor's liquid and gas input flows were regulated and controlled, respectively, using a high-pressure pump and a mass flow controller (MFC). Samples of the product were taken from the condenser after the reactor outflow had passed through the stripper. The hydrogenation reaction was done under 8 bar pressure of H_2_ at 220 °C, the H_2_/HC was considered as 2.51 and the WHSV was 4 for 3gr of catalyst. The reactor’s feedstock (PyGas) composition is shown in Table 1.

**Table 1:** PONA analysis of the PyGas used as feed.

Composition wt%	Paraffin	Olefin	Naphthene	Aromatic	Others	Total
C_3_	0.000				N8 + P8 = 0.000	0.000
C_4_	0.000	0.000				0.000
C_5_	0.670	0.000	0.030		N8 + P9 = 0.000	0.700
C_6_	0.660	4.970	0.470	63.840		69.940
C_7_	0.360	1.600	0.150	15.520	N9 + P9 = 0.000	17.630
C_8_	0.390	0.740	0.180	9.150		C8 + C9 = 11.730
C_9_	0.070	0.240	0.020	0.940		
C_10+_						0.000
Total	2.150	7.550	0.850	89.450	0.000	100.000

### Characterization of the developed catalysts

An S-4700, Hitachi, Krefeld, Germany, microscope equipped with FESEM was used to examine the morphology of the materials. Cu-K radiation (λ = 0.15418 nm) was used in a powder diffractometer (Rigaku TTRAX III, Tokyo, Japan), which was operated at 30 kV and 20 mA, for the XRD. A pace of 4/min was used to gather data on the XRD patterns gathered throughout a range of 5–90 in 2 modes. Flowing nitrogen (60 cm^3^/min) was heated at a rate of 10 °C /min using the thermal stability test method known as thermogravimetric analysis (TGA). It was decided to use a thermogravimetric analyzer (TA Instrument 5100, Dynamic TGA Q500, to quantify any temperature-dependent weight changes in the sample). The ASAP 2020 accelerated surface area and porosity technology were used to evaluate N_2_ adsorption–desorption isotherms at − 196 °C and estimate the samples' surface attributes (Micro metrics, Norcross, GA, USA). For P/P_o_ = 0.05–0.3, the Brunauer–Emmett–Teller (BET) technique was used to calculate the samples' specific surface areas (SSAs). Moreover, the Inductively Coupled Plasma Mass Spectrometry (ICP-MS) or ICP Mass Spectrometry is used to determine the weight percentage of metal and hydrocarbon in synthesized samples.

## Results and discussion

### XRD

**Figure 3:**
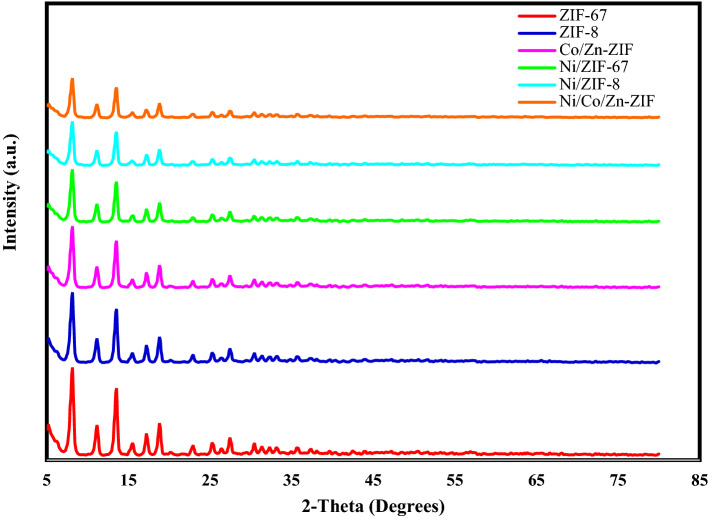
Powder XRD patterns of ZIF-67, ZIF-8, CoZn-ZIF, NiCo-ZIF, NiZn-ZIF and NiCoZn-ZIF.

All the samples showed a similar pattern to their monometallic ZIFs (ZIF-8 and ZIF-67) in Fig. 3. The polymetallic samples including ZnCo-ZIF, ZnNi-ZIF, and ZnCoNi-ZIF showed a slight decrease in their peak intensity due to the lower crystal growth of ZIF. The powder XRD patterns of the as-prepared NiCo-ZIF and NiZn-ZIF are matched with the ZIF-67 and ZIF-8 with a decrease in the peak intensities. There is no sign of Ni, NiO, or any amorphous peaks, indicating that Nickel is embedded in the ZIF structure.

**Figure 4:**
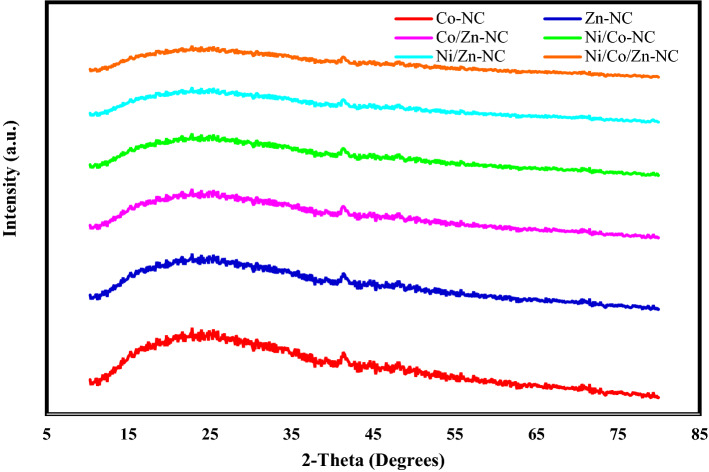
Powder XRD patterns of samples after carbonization process.

After carbonization process, these XRD patterns were entirely changed as shown in Fig. 4. Moreover, XRD peaks were decreased dramatically compare to initial ZIF structures before carbonization.

### N_2_ adsorption/desorption

**Figure 5:**
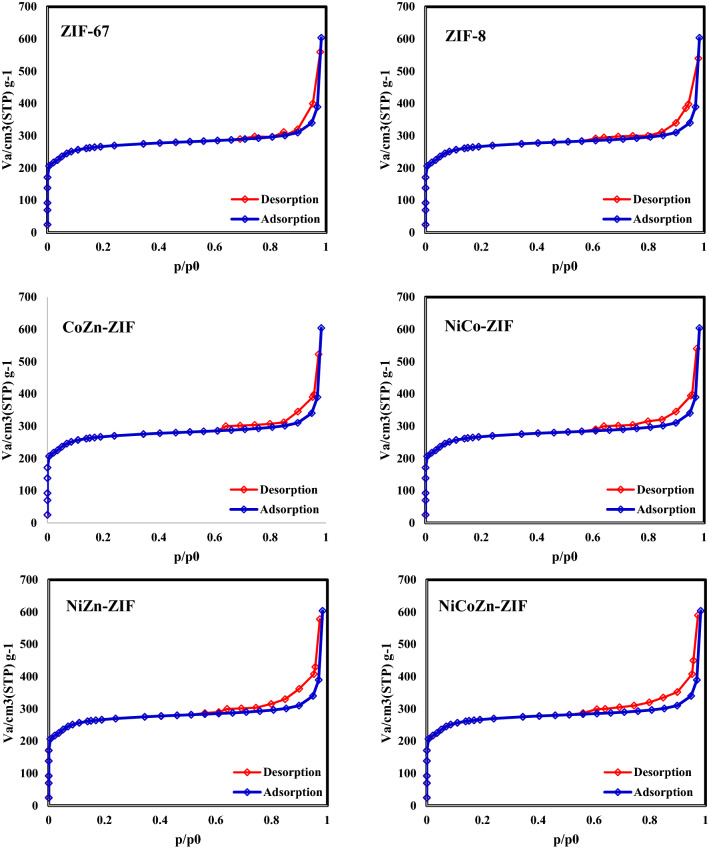
N_2_ adsorption/desorption isotherms of ZIF-67, ZIF-8, CoZn-ZIF, NiCo-ZIF, NiZn-ZIF and NiCoZn-ZIF.

As can be seen in Fig. 5, the N_2_ adsorption/desorption isotherms of all samples are between type-I and type-IV showing the micro and mesoporous structures. The formed hysteresis loops are H4 type which may indicate the presence of narrow slit-like micropores in the material.

**Figure 6:**
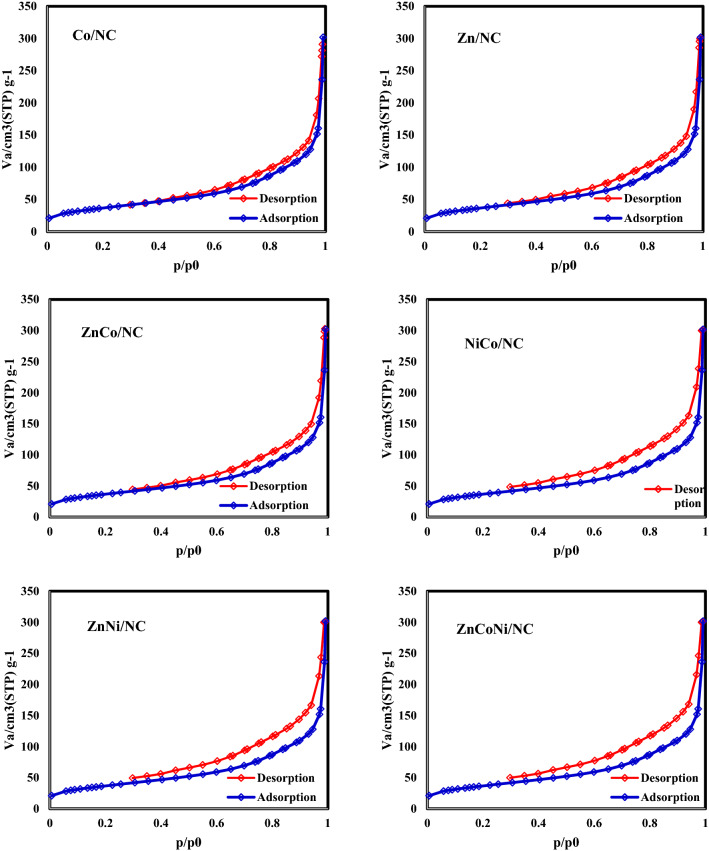
N_2_ adsorption/desorption isotherms of samples after carbonization process.

Moreover, the N2 adsorption/desorption isotherms of samples after carbonization process were shown in Fig. 6. As can be seen, the mesoporous areas which indicates the hysteresis were extended.

BET surface area, average pore diameter, and the total pore volume of the synthesized catalysts are summarized in Table 2. As can be seen, ZIF-67 has the highest surface area with the lowest pore diameter. With the addition of secondary or third metals to the ZIF structures, the surface areas decrease and the pore diameters increase due to the pore blocking.

**Table 2:** BET surface area, average pore diameter, and the total pore volume of ZIF-67, ZIF-8, CoZn-ZIF, NiCo-ZIF, NiZn-ZIF and NiCoZn-ZIF.

Catalyst	ZIF-67	ZIF-8	CoZn-ZIF	NiCo-ZIF	NiZn-ZIF	NiCoZn-ZIF
BET surface area (m^2^ g^−1^)	1247	1135	1036	975	854	587
Average pore diameter (nm)	3.042	3.26	3.56	3.87	4.05	6.49
Total pore volume (cm^3^ g^−1^)	0.95	0.95	0.92	0.90	0.85	0.58

After a carbonization step the N_2_ adsorption/desorption isotherms of all samples turned into type-IV which shows that all samples have become mesoporous due to the destruction of ZIF structure and forming N-doped carbon. The formed hysteresis loops are H3 type which indicates the mesopores are mainly include interparticle pores. Moreover, as you see in Table 3. the average pore diameter of all samples increased. Nevertheless, the BET surface area of all samples still is higher than common catalyst supports like Ɣ-Al_2_O_3_ (~ 100 m^2^/gr).

**Table 3:** BET surface area, average pore diameter, and the total pore volume of Co/NC, Zn/NC, CoZn/NC, NiCo/NC, NiZn/NC and NiCoZn/NC.

Catalyst	Co/NC	Zn/NC	CoZn/NC	NiCo/NC	NiZn/NC	NiCoZn/NC
BET surface area (m^2^ g^−1^)	536.84	492.37	438.26	386.37	346.22	234.64
Average pore diameter (nm)	7.42	8.35	8.65	9.42	9.84	11.42
Total pore volume (cm^3^ g^−1^)	0.57	0.52	0.50	0.49	0.47	0.44

### TGA

**Figure 7:**
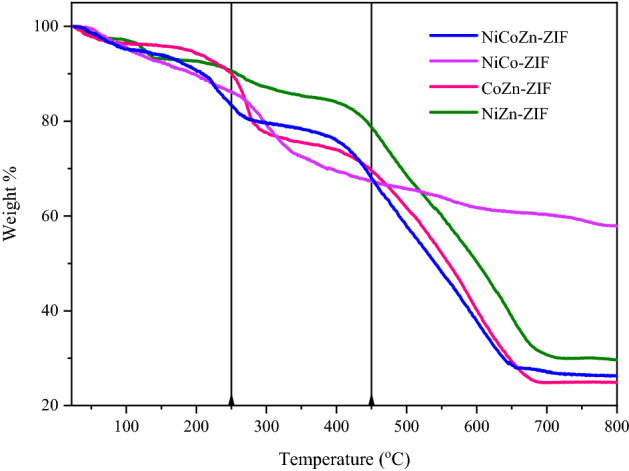
TGA analysis of as prepared NiZn-ZIF, CoZn-ZIF, NiCo-ZIF and NiCoZn-ZIF.

The thermal decomposition point of the as-prepared ZIFs was obtained using TGA (Fig. 7) in which the solvent evaporation from the structure (20 wt%) was observed in the initial range of around 250 °C. The thermal decomposition was initiated at approximately 450 °C for all the samples.

### FE-SEM

**Figure 8:**
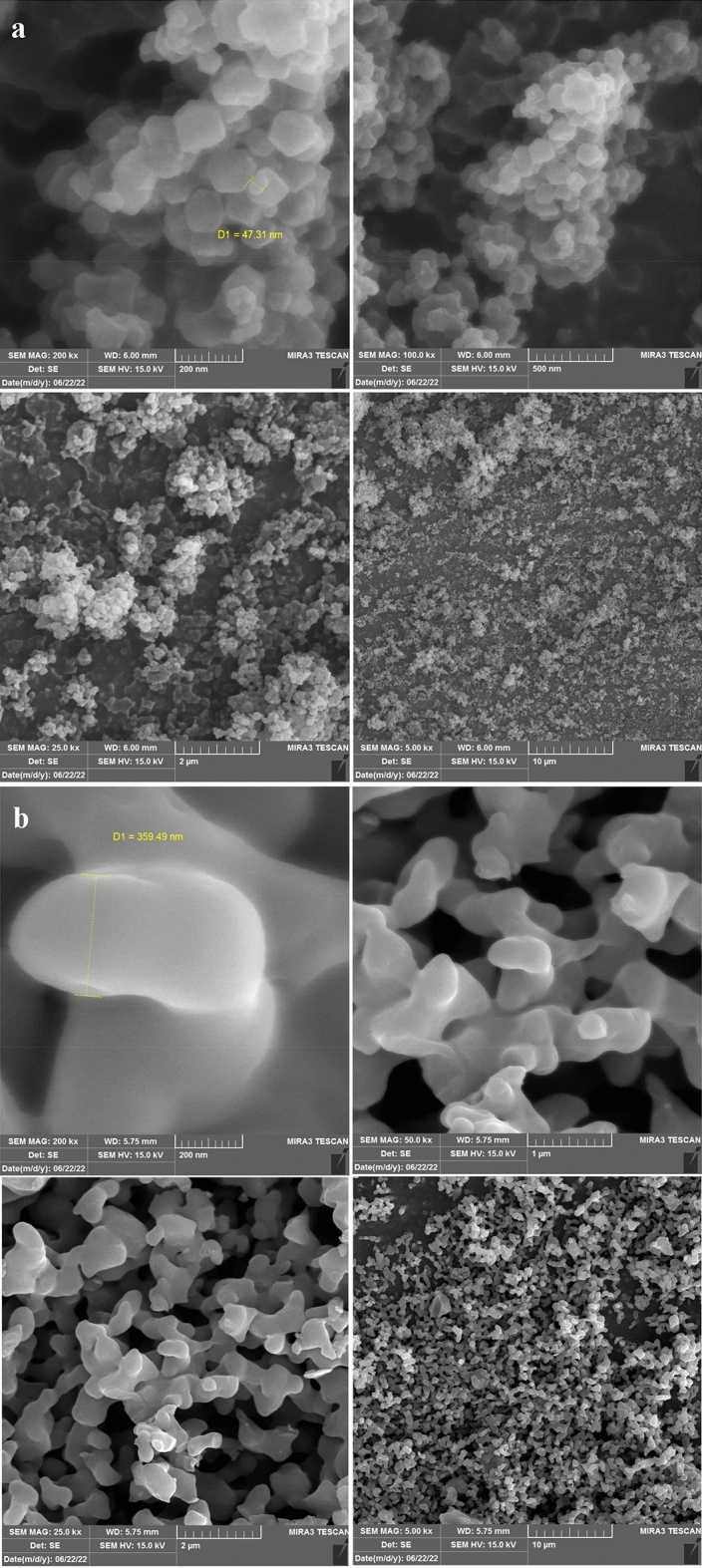
FESEM images of (**a**) NiCoZn-ZIF and (**b**) NiCoZn/NC.

To investigate the bulk-scale morphologies of the composites, we have carried out FE-SEM analysis. The SEM images of the initial NiCoZn-ZIF without the carbonization procedure are shown in Fig. 8. These images reveal that all samples exist of nano-sized crystals which have nearly a rhombic dodecahedron shape. In addition, as can be deduced from these SEM images the average particle size is 100 nm. Moreover, the SEM images of NiCoZn/NC after the carbonization procedure were shown, as can be deduced from these SEM images the average particle size is 400 nm.

### ICP-OES

According to the ICP-OES results shown in Table 4, in single metal ZIFs including ZIF-8 and ZIF-67, the total metal contents are 33% and 34% respectively. In polymetallic samples with Nickel, the Ni content was less than Co and Zn content nevertheless they were used in equal amounts in the synthesis. This could be due to the weaker bond between Ni^2+^ and 2-MeIM compared to Co^2+^ and Zn^2+^ based on hard and soft, acids and bases (HSAB) theory^31^ After the pyrolysis step, the metal content of all samples decreased dramatically.

**Table 4:** ICP-OES results of different ZIFs and their derivatives.

Component	Zn	Co	Ni	Other
ZIF-8	33%	–	–	67%
ZIF-67	–	34%	–	66%
CoZn-ZIF	17%	21%	–	62%
NiCo-ZIF	–	27%	6%	67%
NiZn-ZIF	25%	–	7%	68%
NiCoZn-ZIF	17%	19%	4%	60%
Zn/NC	12%	–	–	88%
Co/NC	–	14%	–	86%
CoZn/NC	7%	9%	–	84%
NiCo/NC		10%	3%	87%
NiZn/NC	8%	–	3%	89%
NiCoZn/NC	5%	7%	2%	86%

### PyGas hydrogenation activity test

Before starting the feeding process with PyGas, the catalysts should be exposed to hydrogen media to activate their metallic sites by the reduction process. This process was done for 2 h at 400 °C and 5 bar.

**Figure 9:**
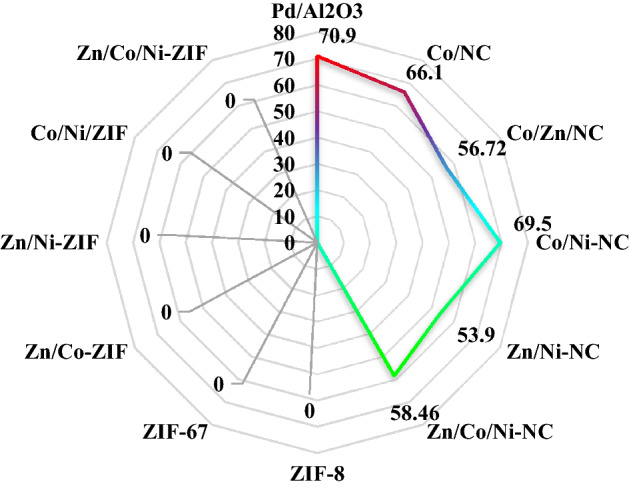
Conversion of total olefins in the hydrogenation reaction.

Figure 9 indicated the conversion of each catalyst in the hydrogenation of PyGas. The commercial Pd/Al_2_O_3_ catalyst showed a 70.9% conversion of total olefins and NiCo/NC showed 69.5% conversion of total olefins which can truly compete with the noble metal commercial Pd/Al_2_O_3_ catalyst. As you can see doping nickel successfully enhanced the hydrogenation properties of Co/NC from 66.1 to 69.5% (NiCo/NC) also the same for CoZn/NC from 56.72 to 58.46% (NiCoZn/NC).

**Figure 10:**
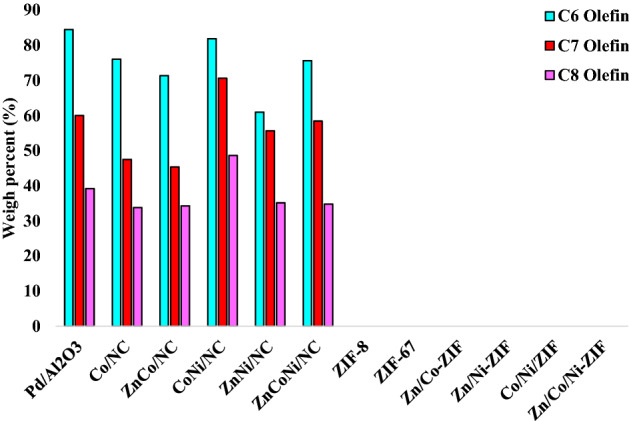
Conversion of C_6_, C_7,_ and C_8_ in the hydrogenation reaction.

Moreover, details of olefins conversions for C_6_, C_7_, and C_8_ were shown in Fig. 10 for each catalyst too. The conversion of Pd/Al_2_O_3_ in the hydrogenation of different olefins for C_6_, C_7_, and C_8_ separately is 85%, 60%, and 39%, respectively.

The decrease of conversion for olefins with a longer carbon chain is due to their more difficult diffusion into the support. The synthesized NiCo/NC catalyst showed a comparable conversion with the commercial catalyst which consists of precious metals like palladium. In addition, the conversion of total olefins decreased, respectively for Co/NC, NiCoZn/NC, CoZn/NC, and NiZn/NC as the active sites decreased. The NiCo/NC showed a lower conversion for C_6_ olefins (81%) than the commercial catalyst. Still, it performed better for C_7_ (70%) and C_8_ (48%) due to the higher BET surface area and more accessible active sites. Co/NC hydrogenated C_6_ olefins like the commercial catalyst (76%) but it showed a setback in C_7_ and C_8_ hydrogenation completely similar to CoZn/NC. But NiZn/NC did the exact opposite it couldn't have a proper conversion in the hydrogenation of C_6_ (61%) in comparison to the commercial catalyst, nevertheless it did well in the hydrogenation of C_7_ (55%) and C_8_ (35%). NiCoZn/NC had an average performance for all three types of olefins. Various kinds of pristine ZIFs did not have any conversion, because the metal content in the pristine ZIFs is in the ion phase in a coordinative bond with the 2-MIM and it cannot work as a metallic active site.

## Conclusions

In this study, new nanostructure catalysts derived from zeolitic metal–organic framework namely ZIF-8 and ZIF-67 were used to investigate their hydrogenation capability. Owning to its great hydrogenation capability of Nickle, the structures of the ZIF-8 and ZIF-67 were improved by Nickle through in situ synthesis. Afterward, a pyrolysis step is designed to turn the intrinsic metal ions of ZIF-8 and ZIF-67 into metallic nanoparticles besides turning the 2-MIM ligand into the electroconductive N-doped carbon material as a support. PyGas hydrogenation reaction was chosen as an important industrial process in olefin units to evaluate the performance of prepared catalysts. Characterization tests confirmed the credit of our synthesizing process. It showed that by the addition of secondary or third metals to the ZIF structures, the surface areas decrease and the pore diameters increase up to 6.5 nm. Also the pyrolysis step had the same effect and increased the pore diameters up to 11.42 nm due to the destruction of ZIF structure and formation of N-doped carbon materials.

Moreover, it can be figured out from the hydrogenation results that Zinc has not a significant impact on the hydrogenation reaction. The catalysts with Cobalt species had a better conversion for C_6_ olefins and the catalysts which had Nickel species were more proper for C_7_ and C_8_ olefins. Consequently, polymetallic ZIFs, especially, NiCo/NC could be a competitive catalyst for hydrogenation of PyGas by 69.5% conversion of olefins compared to the noble metal commercial catalysts like Pd/Al_2_O_3_by 70.9% conversion of olefins.

## Data Availability

The datasets used and/or analyzed during the current study available from the corresponding author on reasonable request.

## References

[1] Larriba M (2017). Extraction of aromatic hydrocarbons from pyrolysis gasoline using tetrathiocyanatocobaltate-based ionic liquids: Experimental study and simulation. Fuel Process. Technol..

[2] Navarro P (2019). Dearomatization of pyrolysis gasoline by extractive distillation with 1-ethyl-3-methylimidazolium tricyanomethanide. Fuel Process. Technol..

[3] Zhou Z, Zeng T, Cheng Z, Yuan W (2010). Preparation of a catalyst for selective hydrogenation of pyrolysis gasoline. Ind. Eng. Chem. Res..

[4] Gaspar AB, dos Santos GR, de Souza Costa R, da Silva MAP (2008). Hydrogenation of synthetic PYGAS—Effects of zirconia on Pd/Al_2_O_3_. Catal. Today.

[5] Yang Z, Han J, Fan Q, Jia H, Zhang F (2018). Catalytic hydrogenation of a pyrolysis gasoline model feed over supported NiRu bimetallic catalysts with Ru content from 0.01 wt% to 0.1 wt%. Appl. Catal. A Gen..

[6] Zhou Z, Zeng T, Cheng Z, Yuan W (2011). Diffusion-enhanced hierarchically macro-mesoporous catalyst for selective hydrogenation of pyrolysis gasoline. AIChE J..

[7] Enache DI, Landon P, Lok CM, Pollington SD, Stitt EH (2005). Direct comparison of a trickle bed and a monolith for hydrogenation of pyrolysis gasoline. Ind. Eng. Chem. Res..

[8] Wen X, Li R, Yang Y, Chen J, Zhang F (2013). An egg-shell type Ni/Al_2_O_3_ catalyst derived from layered double hydroxides precursor for selective hydrogenation of pyrolysis gasoline. Appl. Catal. A Gen..

[9] Hoffer BW (2004). Enhancing the start-up of pyrolysis gasoline hydrogenation reactors by applying tailored ex situ presulfided Ni/Al_2_O_3_ catalysts. Fuel.

[10] Betti C (2012). Effect of the sequence of impregnation on the activity and sulfur resistance of Pt–Ni/γ-Al_2_O_3_ bimetallic catalysts for the selective hydrogenation of styrene. Appl. Catal. A Gen..

[11] Qian Y (2011). Enhancement of pyrolysis gasoline hydrogenation over Zn-and Mo-promoted Ni/γ-Al_2_O_3_ catalysts. Catal. Commun..

[12] Xiang J, Wen X, Zhang F (2014). Supported nickel–cobalt bimetallic catalysts derived from layered double hydroxide precursors for selective hydrogenation of pyrolysis gasoline. Ind. Eng. Chem. Res..

[13] Ferrando R, Jellinek J, Johnston RL (2008). Nanoalloys: From theory to applications of alloy clusters and nanoparticles. Chem. Rev..

[14] Liu X, Wang D, Li Y (2012). Synthesis and catalytic properties of bimetallic nanomaterials with various architectures. Nano Today.

[15] Munoz-Flores BM, Kharisov BI, Jiménez-Pérez VM, Elizondo Martínez P, Lopez ST (2011). Recent advances in the synthesis and main applications of metallic nanoalloys. Ind. Eng. Chem. Res..

[16] Huang Y, Sachtler WMH (1999). Catalytic hydrogenation of nitriles over supported mono-and bimetallic catalysts. J. Catal..

[17] Braos-García P, García-Sancho C, Infantes-Molina A, Rodríguez-Castellón E, Jiménez-López A (2010). Bimetallic Ru/Ni supported catalysts for the gas phase hydrogenation of acetonitrile. Appl. Catal. A Gen..

[18] Yang Y, Gao G, Zhang X, Li F (2014). Facile fabrication of composition-tuned Ru–Ni bimetallics in ordered mesoporous carbon for levulinic acid hydrogenation. ACS Catal..

[19] Murugesan K (2018). Cobalt-based nanoparticles prepared from MOF–carbon templates as efficient hydrogenation catalysts. Chem. Sci..

[20] Guo M, Zhang M, Liu R, Zhang X, Li G (2022). State-of-the-art advancements in photocatalytic hydrogenation: Reaction mechanism and recent progress in metal-organic framework (MOF)-based catalysts. Adv. Sci..

[21] Sadjadi S, Koohestani F (2022). Palladated composite of Cu-BDC MOF and perlite as an efficient catalyst for hydrogenation of nitroarenes. J. Mol. Struct..

[22] Li Z (2022). Mesoporous silica stabilized MOF nanoreactor for highly selective semi-hydrogenation of phenylacetylene via synergistic effect of Pd and Ru single site. Nano Res..

[23] Wang S (2022). Highly efficient hydrogenation of phenol to cyclohexanol over Ni-based catalysts derived from Ni-MOF-74. React. Chem. Eng..

[24] Dai H (2021). Recent advances on ZIF-8 composites for adsorption and photocatalytic wastewater pollutant removal: Fabrication, applications and perspective. Coord. Chem. Rev..

[25] Lai Z (2018). Development of ZIF-8 membranes: Opportunities and challenges for commercial applications. Curr. Opin. Chem. Eng..

[26] Su T, Lu G-P, Sun K-K, Zhang M, Cai C (2022). ZIF-derived metal/N-doped porous carbon nanocomposites: Efficient catalysts for organic transformations. Catal. Sci. Technol..

[27] Zhong G, Liu D, Zhang J (2018). The application of ZIF-67 and its derivatives: Adsorption, separation, electrochemistry and catalysts. J. Mater. Chem. A.

[28] Butova VV, Budnyk AP, Bulanova EA, Lamberti C, Soldatov AV (2017). Hydrothermal synthesis of high surface area ZIF-8 with minimal use of TEA. Solid State Sci..

[29] Lee JG (2020). Structural evolution of ZIF-67-derived catalysts for furfural hydrogenation. J. Catal..

[30] Zhao L (2020). ZIF-67 derived Co, Fe, Ni co-doped porous carbon as an efficient electrocatalyst for hydrogen evolution reaction. J. Porous Mater..

[31] Pearson RG (1968). Hard and soft acids and bases, HSAB, part 1: Fundamental principles. J. Chem. Educ..

